# Optical Coherence Tomography Angiography of Nonarteritic Cilioretinal Artery Occlusion Alone

**DOI:** 10.1155/2021/8845972

**Published:** 2021-01-27

**Authors:** Toyo Ikebukuro, Tsutomu Igarashi, Shuhei Kameya, Takeshi Arima, Tomoyuki Kunishige, Hiroshi Takahashi

**Affiliations:** ^1^Department of Ophthalmology, Nippon Medical School, 1-1-5 Sendagi, Bunkyo-ku, Tokyo, Japan; ^2^Department of Ophthalmology, Nippon Medical School Chiba Hokusoh Hospital, 1715 Kamagari, Inzai, Chiba, Japan

## Abstract

Cilioretinal artery occlusion (CLRAO) is a rare disease. Here, we report the case of a 70-year-old man with nonarteritic cilioretinal artery occlusion alone. The patient was allergic to fluorescein. Therefore, we followed the retinal circulation with optical coherence tomography angiography (OCTA). OCTA at 40 days postonset showed partial improvement in the retinal circulation.

## 1. Introduction

The retinal artery is a terminal artery responsible for blood flow in the inner retinal layer. Retinal artery occlusion (RAO) causes pallor and edematous changes in the retina and thickening of the inner retinal layer in the ischemic area. Basically, the central retinal artery plays a major role in retinal circulation, but this circulation is diverse. Cilioretinal arteries, which belong to the posterior ciliary artery system, usually arise from the peripapillary choroid or directly from one of the short posterior ciliary arteries. In a previous study, the incidence of cilioretinal arteries in 2,000 eyes of 1,000 people was evaluated by fundus photographs and fluorescein angiography. Among them, 959 people were patients of the department of ophthalmology, Baylor College of Medicine. Forty-one people were healthy young adult volunteers with no history of ophthalmic disease. Cilioretinal arteries were reported to be present in 49.5% of these people (34.9% in one eye only and 14.6% in both eyes) [1]. Cilioretinal artery occlusion (CLRAO) is a rare disease, occurring in only about 5% of RAO cases [2]. To date, fluorescein angiography (FA) has been a standard method for the diagnosis of RAO. However, recently, noninvasive microcirculation evaluation by optical coherence tomography angiography (OCTA) has become possible [3]. Here, we report a case of CLRAO that was followed up with OCTA because of an allergic reaction to fluorescein.

## 2. Case Presentation

We report a case of CLRAO in a 70-year-old male. He became aware of loss of vision in his left eye upon waking at 07:00 on November 14, 2018. He had no subjective symptoms when he went to bed at 23:00 the night before. After visiting a nearby eye clinic, he was referred to our hospital because of suspected left RAO. He arrived at our hospital at 15:00; thus, it was estimated that 8–16 hours had elapsed since the onset of RAO.

The patient had no history of risk factors for RAO, such as hypertension, hypercholesterolemia, or diabetes mellitus, and no family history of ophthalmologic disease, but had a history of smoking about 20 cigarettes a day. His visual acuity at the initial visit was 20/16 in the right eye and 20/40 in the left eye. On slit-lamp examination, no abnormalities were found in the anterior segment of the eye. Intraocular pressure was 17.0 mmHg in the right eye and 16.5 mmHg in the left eye.

**Figure 1:**
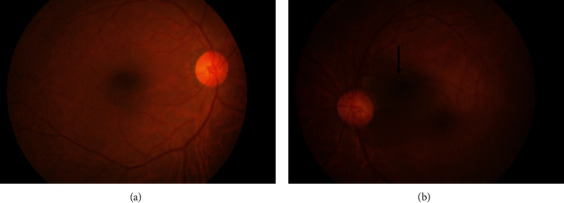
Color fundus photographs of the (a) right eye and (b) left eye. (a) In the right eye, no obvious fundus abnormalities are apparent. (b) In the left eye, the cilioretinal artery extends from the upper temporal side of the optic nerve papillae to the temporal side. White change is seen in the retina along the cilioretinal artery.

Fundus examination showed no abnormalities in the color tone of the optic nerve papillae, and the cup-to-disc ratio was 0.5 in both eyes. No abnormal findings were observed in the retina of the right eye, while a band-like white change was found in the superior macular area and in the superior temporal area in the left eye. The cilioretinal artery extended from the superior temporal side of the optic nerve papillae. The area of white change was consistent with the area of the cilioretinal artery, and therefore, CLRAO was suspected (Figure 1).

**Figure 2:**
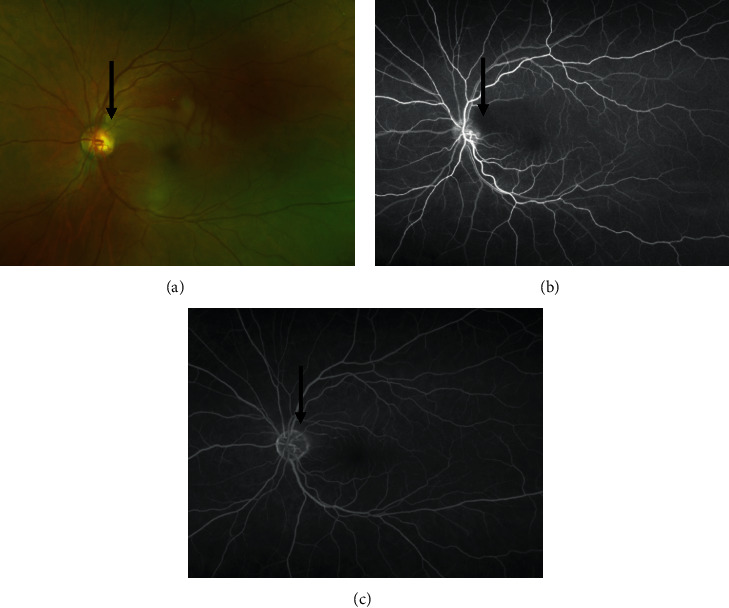
Fluorescein angiography (FA). (a) The left ocular fundus photograph. As in the fundus examination, the image shows a cilioretinal artery originating from the optic nerve papillae. White changes (arrow) are seen in the retina around the cilioretinal artery. (b) The image 20 s after the start of FA. Fluorescein staining of the cilioretinal artery was delayed (arrow). Subsequently, the cilioretinal artery was gradually refluxed from the distal side. (c) The image at 2 min and 39 s after the start of FA. The area around the cilioretinal artery was stained.

**Figure 3:**
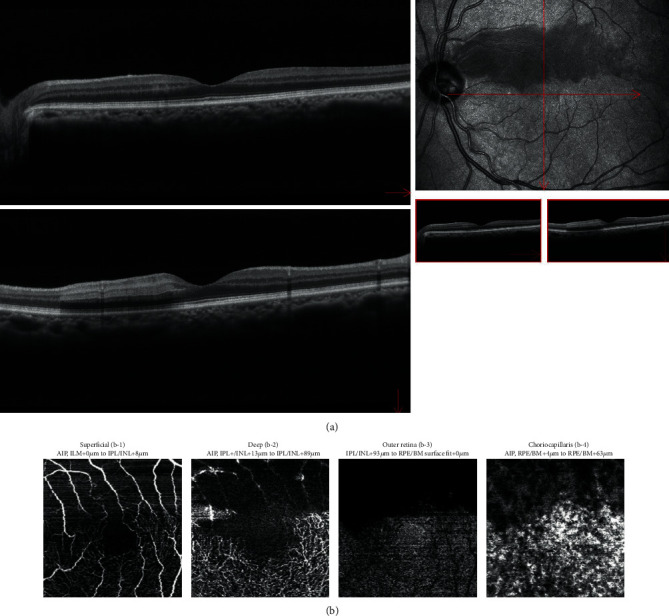
(a) Optical coherence tomography (OCT) and (b) optical coherence tomography angiography (OCTA) (onset): (b-1) superficial layer, (b-2) deep layer, (b-3) outer retina layer, and (b-4) choriocapillaris layer of OCTA. (a) OCT shows a thickening of the inner retinal layer consistent with the white change on the fundus photograph. On infrared reflectance images, the area of white change in the fundus photograph is depicted with low luminance, and the area of ischemia can be seen more clearly. (b) In OCTA, the flow sign is decreased in all layers.

Then, FA and optical coherence tomography (OCT) and OCTA (RS-3000 Advance 2; NIDEK Co., Ltd., Aichi, Japan) were performed. After confirming that there were no problems with the blood test, the patient underwent FA using ultrawide-field retinal imaging device (OPTOS California; Nikon Healthcare Japan Inc., Tokyo, Japan). The results showed delayed reflux in the cilioretinal artery of the left eye (Figure 2). After FA, although no abnormal vital signs were seen, a rash was found in the patient's chest, suggesting an allergic reaction to fluorescein. Therefore, OCTA was used to follow up his clinical course thereafter. OCT showed a thickening of the inner retinal layer consistent with the area of white change (Figure 3(a)). In infrared reflectance (IR) images, the area of white change in the fundus photograph was depicted with low luminance, and the area of ischemia could be seen more clearly. In OCTA, the flow sign in the superior macular white change area was reduced in the superficial retinal layer (Figure 3(b)). Due to edema of the inner layer of the retina, the flow signs of the deep, outer retina and choriocapillaris were unclear.

The patient was admitted to our hospital on the same day. After confirming no intracranial abnormalities by computed tomography, 240,000 units of urokinase-type plasminogen activator were administered per day for 3 days. The patient was also scheduled to receive alprostadil (prostaglandin E1) for 3 days; however, it caused vascular pain on the first day and was therefore stopped. Hyperbaric oxygen therapy could not be performed because of a history of spontaneous pneumothorax. Stellate ganglion block was performed five times by an anesthesiologist from the fourth day after onset.

**Figure 4:**
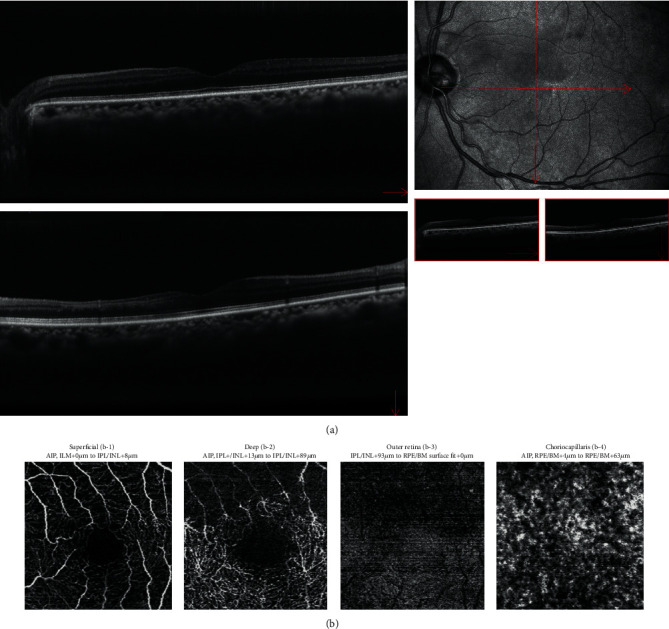
(a) OCT and (b) OCTA at 40 days from disease onset: (b-1) superficial layer, (b-2) deep layer, (b-3) outer retina layer, and (b-4) choriocapillaris layer of OCTA. (a) OCT shows thinning of the inner retinal layer consistent with the white change on the fundus photograph. (b) OCTA shows a decrease in the flow sign. Compared with at disease onset, the flow sign is improved in all layers, but the improvement is mainly in the superficial layer.

**Figure 5:**
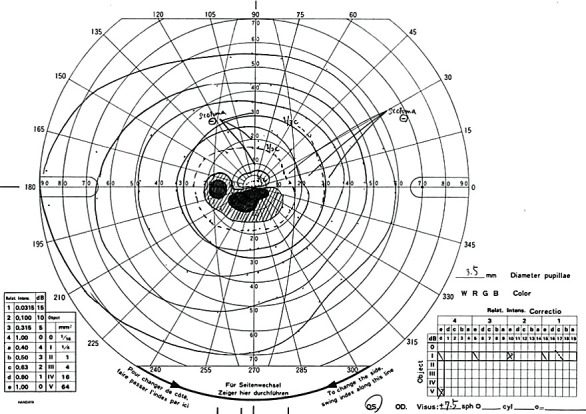
Visual field test by Goldmann perimetry at 56 days from disease onset. A scotoma is seen from slightly below center to the ear side. The location of this paracentral scotoma coincides with the location of cilioretinal artery occlusion.

Magnetic resonance angiography and ultrasonography of the carotid artery were performed to investigate cerebrovascular and carotid artery lesions, but no disease causing CLRAO was found. On day 5, oral kallidinogenase was started, and his visual acuity gradually improved. On day 40, his left visual acuity eye was restored to 20/25, and OCT showed thinning of the lesion that had the white change. In OCTA, the flow sign was improved in all layers compared to that on day 1 (Figure 4). On day 56, visual field test showed a paracentral scotoma (Figure 5).

## 3. Discussion

In a previous study evaluating 2,000 eyes in 1,000 people using color fundus photography and FA, a cilioretinal artery was found in 49.5% of all patients and 32.1% of all eyes [1]. In the present case, a cilioretinal artery related to macular circulation was found in the left eye only. RAO is a rare disease, with an incidence of 1–10 per 100,000 population. Symptomatic CLRAO is even rarer, reported in 5.3–7.1% of all RAO cases [2].

CLRAO can be classified clinically into three categories: (1) nonarteritic CLRAO alone, (2) arteritic CLRAO associated with giant cell arteritis, and (3) CLRAO associated with central retinal vein occlusion [4]. The present case corresponds to nonarteritic CLRAO alone.

In the present case, visual acuity was 20/25 at 40 days after disease onset. The cilioretinal artery was occluded, resulting in ischemic changes in only the upper macula (the reflux area). Since this was partial ischemia of the macula, vision loss was considered to be mild. Important causes of RAO include hypertension, diabetes mellitus, vascular lesions, and a history of smoking. The risk factor in this patient was a history of smoking. Regarding treatment, hyperbaric oxygen therapy was not possible, but urokinase drip and stellate ganglion block could be administered. However, an allergic reaction to fluorescein made it impossible to follow up using FA. Therefore, OCTA was used to assess blood flow. On day 1, a decrease in the flow sign in all layers was observed in the ischemic area. On day 40, the flow sign had considerably improved in the superficial layer, the outer retina, and the choriocapillaris layers, while in the deep layer, although some improvement of blood flow was observed in areas close to the macula, no significant improvement in the flow sign was seen.

Abdellah reported that OCTA results in 11 cases of CRAO showed decreased flow in both the superficial and deep layers, but the superficial layer was more injured [5]. In the present case, there was more damage in the deep layer, which may suggest that CLRAO may be more damaging to the capillary network in the deep layer than in the superficial layer. However, this is only a case report and more cases are needed.

One disadvantage of OCTA is the effect of artifacts. The outer retina and choriocapillaris layers are nourished by choroidal vessels. Therefore, it is unlikely that retinal artery occlusion would cause circulatory failure in these layers. Decreased flow sign in the outer retina and choriocapillaris layers may be due to artifacts caused by edematous changes in the inner retinal layer.

Infrared reflectance (IR) images showed a hyporeflectant area clearly suggestive of ischemia that was consistent with the white change seen on the fundus examination and FA. Findings on IR images may therefore assist in the evaluation of ischemic areas in the acute phase of RAO.

Although susceptible to artifacts, OCTA is superior to FA in imaging in a patient with fluorescein allergy in cilioretinal artery occlusion.
